# Supplementary material for: OpenMM 7: Rapid development of high performance algorithms for molecular dynamics
Source: PLoS Comput Biol. 2017 Jul 26;13(7):e1005659. doi: 10.1371/journal.pcbi.1005659 (PMC5549999; doi:10.1371/journal.pcbi.1005659)
Supplement: S1 Source Code — (ZIP) [file pcbi.1005659.s002.zip › OpenMM-7.0.1-Source/docs/api-python/generated/simtk.openmm.app.statedatareporter.StateDataReporter.html]

StateDataReporter — OpenMM 7.0.1.dev-5e86c4f documentation


# StateDataReporter¶

*class* `simtk.openmm.app.statedatareporter.``StateDataReporter`(*file*, *reportInterval*, *step=False*, *time=False*, *potentialEnergy=False*, *kineticEnergy=False*, *totalEnergy=False*, *temperature=False*, *volume=False*, *density=False*, *progress=False*, *remainingTime=False*, *speed=False*, *elapsedTime=False*, *separator='*, *'*, *systemMass=None*, *totalSteps=None*)¶
:   StateDataReporter outputs information about a simulation, such as energy and temperature, to a file.

    To use it, create a StateDataReporter, then add it to the Simulation’s list of reporters. The set of
    data to write is configurable using boolean flags passed to the constructor. By default the data is
    written in comma-separated-value (CSV) format, but you can specify a different separator to use.

    `__init__`(*file*, *reportInterval*, *step=False*, *time=False*, *potentialEnergy=False*, *kineticEnergy=False*, *totalEnergy=False*, *temperature=False*, *volume=False*, *density=False*, *progress=False*, *remainingTime=False*, *speed=False*, *elapsedTime=False*, *separator='*, *'*, *systemMass=None*, *totalSteps=None*)¶
    :   Create a StateDataReporter.

        |  |  |
        | --- | --- |
        | Parameters: | - **file** (*string or file*) – The file to write to, specified as a file name or file object - **reportInterval** (*int*) – The interval (in time steps) at which to write frames - **step** (*bool=False*) – Whether to write the current step index to the file - **time** (*bool=False*) – Whether to write the current time to the file - **potentialEnergy** (*bool=False*) – Whether to write the potential energy to the file - **kineticEnergy** (*bool=False*) – Whether to write the kinetic energy to the file - **totalEnergy** (*bool=False*) – Whether to write the total energy to the file - **temperature** (*bool=False*) – Whether to write the instantaneous temperature to the file - **volume** (*bool=False*) – Whether to write the periodic box volume to the file - **density** (*bool=False*) – Whether to write the system density to the file - **progress** (*bool=False*) – Whether to write current progress (percent completion) to the file.   If this is True, you must also specify totalSteps. - **remainingTime** (*bool=False*) – Whether to write an estimate of the remaining clock time until   completion to the file. If this is True, you must also specify   totalSteps. - **speed** (*bool=False*) – Whether to write an estimate of the simulation speed in ns/day to   the file - **elapsedTime** (*bool=False*) – Whether to write the elapsed time of the simulation in seconds to   the file. - **separator** (*string=’,’*) – The separator to use between columns in the file - **systemMass** (*mass=None*) – The total mass to use for the system when reporting density. If   this is None (the default), the system mass is computed by summing   the masses of all particles. This parameter is useful when the   particle masses do not reflect their actual physical mass, such as   when some particles have had their masses set to 0 to immobilize   them. - **totalSteps** (*int=None*) – The total number of steps that will be included in the simulation.   This is required if either progress or remainingTime is set to True,   and defines how many steps will indicate 100% completion. |

    Methods

    |  |  |
    | --- | --- |
    | `__init__`(file, reportInterval[, step, time, ...]) | Create a StateDataReporter. |
    | `describeNextReport`(simulation) | Get information about the next report this object will generate. |
    | `report`(simulation, state) | Generate a report. |

    `__delattr__`¶
    :   x.\_\_delattr\_\_(‘name’) <==> del x.name

    `__format__`()¶
    :   default object formatter

    `__getattribute__`¶
    :   x.\_\_getattribute\_\_(‘name’) <==> x.name

    `__hash__`¶

    `__reduce__`()¶
    :   helper for pickle

    `__reduce_ex__`()¶
    :   helper for pickle

    `__repr__`¶

    `__setattr__`¶
    :   x.\_\_setattr\_\_(‘name’, value) <==> x.name = value

    `__sizeof__`() → int¶
    :   size of object in memory, in bytes

    `__str__`¶

    `describeNextReport`(*simulation*)¶
    :   Get information about the next report this object will generate.

        |  |  |
        | --- | --- |
        | Parameters: | **simulation** (*Simulation*) – The Simulation to generate a report for |
        | Returns: | A five element tuple. The first element is the number of steps until the next report. The remaining elements specify whether that report will require positions, velocities, forces, and energies respectively. |
        | Return type: | tuple |

    `report`(*simulation*, *state*)¶
    :   Generate a report.

        |  |  |
        | --- | --- |
        | Parameters: | - **simulation** (*Simulation*) – The Simulation to generate a report for - **state** (*State*) – The current state of the simulation |

High performance molecular simulation on GPUs


### Navigation

- Application Layer
  - Loaders and Setup
  - Representation and Manipulation
  - Simulation
  - Reporting Output
  - Extras
- Library Layer

©2015, Stanford University and the Authors.
|
Powered by Sphinx 1.3.1
& Alabaster 0.7.7
|
Page source
